# The role of cGMP signalling in regulating life cycle progression of *Plasmodium*

**DOI:** 10.1016/j.micinf.2012.04.011

**Published:** 2012-08

**Authors:** Christine S. Hopp, Paul W. Bowyer, David A. Baker

**Affiliations:** Faculty of Infectious and Tropical Diseases, London School of Hygiene and Tropical Medicine, Keppel Street, London WC1E 7HT, UK

**Keywords:** Malaria, Gametocyte, Schizont, Merozoite, Kinase, Egress

## Abstract

The 3′-5′-cyclic guanosine monophosphate (cGMP)-dependent protein kinase (PKG) is the main mediator of cGMP signalling in the malaria parasite. This article reviews the role of PKG in *Plasmodium falciparum* during gametogenesis and blood stage schizont rupture, as well as the role of the *Plasmodium berghei* orthologue in ookinete differentiation and motility, and liver stage schizont development. The current views on potential effector proteins downstream of PKG and the mechanisms that may regulate cyclic nucleotide levels are presented.

## 1 Guanylyl cyclases and cGMP-phosphodiesterases

The intracellular second messenger cGMP regulates downstream targets, thereby influencing diverse cellular functions, ranging from smooth muscle contractility [1], to retinal phototransduction [2]. cGMP is synthesised by guanylyl cyclases (GCs) and hydrolysed by phosphodiesterases (PDEs). In mammalian cells, natriuretic peptides ANP, BNP and CNP activate membrane-bound GCs, while the soluble GC is believed to be the main intracellular receptor of nitric oxide (NO) [3]. Signalling mechanisms upstream of malaria parasite GCs and the identity of potential membrane receptors that may be able to react to environmental cues and initiate intracellular signalling, remain largely unknown. It is possible that rather than being coupled to receptors, the membrane-bound GCs themselves may be able to receive an extra- or intracellular signal. In *Plasmodium*, there is no soluble GC and the membrane-bound GCs do not appear to contain an NO-binding domain and so it is unclear, whether NO impacts on cGMP signalling in the parasite.

Due to evolutionary early branching, the apicomplexan components of cGMP signalling are much diverged from the mammalian enzymes. In *Plasmodium falciparum*, two membrane-associated GC isoforms (PfGCα and PfGCβ) have been identified [4]. These are unusual predicted bifunctional enzymes with up to 22 transmembrane domains; comprising an N-terminal P-type ATPase-like domain (resembling a calcium pump) and a paired C-terminal GC domain that has a similar topology to mammalian G protein coupled adenylyl cyclases [4,6]. Recombinant expression of the two catalytic domains of PfGCβ demonstrated guanylyl cyclase activity, but no adenylyl cyclase activity [4]. Low levels of PfGCα mRNA expression have been detected in sexual and asexual blood stages by microarray analysis [5,7]. Immunoelectron microscopy using an antibody raised to the catalytic domain of PfGCα suggested that the enzyme may be located in the parasitophorous vacuole membrane (PVM) or the plasma membrane of gametocytes [4]. GCα orthologues from *P. falciparum* and *Plasmodium berghei* are both refractory to deletion and are therefore likely to have an essential function in the development of erythrocytic asexual stages [4,8]. PfGCβ is transcribed at relatively high levels in mature gametocytes, but not in asexual blood stages. The gene was successfully disrupted implying no essential function in the blood stages. PfGCβ-ko lines also exhibited no significant effects on the development of gametocytes or gametogenesis [8], consistent with results obtained by deleting the *P. berghei* orthologue [9].

In *P. falciparum*, four genes encode cyclic nucleotide PDEs (*PfPDEα*, *PfPDEβ*, *PfPDEγ* and *PfPDEδ*). PfPDEα is expressed as two alternatively spliced isoforms (PfPDEαA and PfPDEαB). All four PfPDEs are predicted to comprise 4–6 transmembrane domains, consistent with the finding that most of the PDE activity is present in crude membrane fractions of blood stage parasites [10,11]. This is in contrast to mammalian cells, where few PDEs contain a transmembrane domain [12]. There is biochemical and genetic evidence that PfPDEα and PfPDEδ hydrolyse cGMP [8,10,11,13]. Deletion of *PfPDEα* showed that it is not an essential gene in blood stages, since parasites had no aberrant phenotype. However, cGMP-hydrolysing activity in *PfPDEα*-ko parasites was reduced by 20%, while cAMP-hydrolysis was unaffected [11], consistent with data showing that the catalytic domain of PfPDEα as a recombinant protein has cGMP-PDE activity [10,11]. These data also suggest that at least one other PDE breaks down cGMP in *P. falciparum* blood stages. The most plausible candidate is PfPDEβ, since this is the only other PfPDE highly expressed in blood stages [14]. High levels of cAMP-specific PDE activity have been measured in the asexual blood stages of *P. falciparum*, suggesting that PfPDEβ may be a dual specificity phosphodiesterase [11,15]. PfPDEγ mRNA expression is detectable at low levels in asexual blood stage parasites [11], but was successfully knocked out in *P. falciparum*, without obvious morphological effects in asexual or sexual blood stages [8]. PfPDEδ mRNA is expressed at high levels in gametocytes and while a knockout was also possible, indicating a non-essential role in asexual stages, severe effects on gametogenesis and a 50% reduction of cGMP-PDE activity were observed [8].

While the cGMP-dependent protein kinase (PKG) is believed to be the main target of cGMP, mammalian cells have other proteins that are directly regulated by cGMP, such as cyclic nucleotide-gated ion channels and proteins containing a small molecule and nucleotide binding GAF domain, which in some cases binds to cGMP [16]. *Plasmodium* genomes, each have a single gene encoding PKG, but no cGMP-responsive ion channel was identified [17,18]. An additional protein has been identified (PlasmoDB identifier: PF14_0173, PBANKA_102530) with five putative cyclic nucleotide binding domains, of which the most C-terminal domain is predicted to have cGMP-specificity [13]. The predicted >400 kDa protein is conserved among apicomplexan parasites and its expression has been detected in *P. falciparum* [19,20]. No conserved effector function can be annotated, but interestingly the protein has homology with reticulocyte binding proteins. The remainder of this review will focus on the only characterised cGMP regulated protein in *Plasmodium*, PKG.

## 2 The apicomplexan cGMP-dependent protein kinase

Two genes encode mammalian PKG, type I and II, and two PKG I isoforms are generated by alternative splicing (PKG-Iα and PKG-Iβ) [21]. PKG-I is cytosolic, whereas PKG-II undergoes myristoylation at an N-terminal glycine residue that facilitates anchoring to the plasma membrane [21]. Mammalian PKG is a homodimer, with each monomer comprising both a regulatory and a catalytic domain. An autoinhibitory domain, that is part of the regulatory subdomain of PKG, interacts with and inhibits the catalytic subunit in the absence of cGMP. Upon cGMP-binding to the regulatory domain, a conformational change releases the catalytic domain, leading to activation of the kinase [21,22]. No 3D structural data exist for full length PKG to date, however, crystallographic data are available for the leucine zipper and GKAP docking site of PKG-Iβ [23] and the N-terminal cyclic nucleotide binding domain [24].

The apicomplexan parasites *Toxoplasma gondii* and *Eimeria tenella* both have a single PKG gene copy, but express two isoforms as a result of alternative translation start sites. The shorter isoform localises to the cytosol, while the full length protein undergoes N-terminal myristoylation and palmitoylation, reminiscent of the mammalian PKG II [18]. The single copy *P. falciparum* PKG (PfPKG; PlasmoDB identifier PF14_0346) lacks the consensus motifs for N-terminal myristoylation and palmitoylation [25]. There are structural features, distinct from mammalian cells that are conserved across the apicomplexan PKGs. First, they lack the N-terminal leucine zipper motif present in mammalian PKG, which is thought to mediate homodimerisation, as well as interaction with other proteins, including some substrates. Evidence suggests that the apicomplexan PKGs are monomeric, in contrast to the mammalian isoforms [18]. Interestingly, the *P. falciparum* PKG does have a motif towards the N-terminus of the protein that resembles the pseudosubstrate sequence of mammalian autoinhibitory domains [26], although a regulatory role for this has not been demonstrated.

Another distinction concerns the number of cGMP-binding sites in the regulatory domain. Mammalian PKGs contain two cGMP-binding sites whereas apicomplexan isoforms contain an additional, third, cGMP-binding site and a degenerate fourth which lacks key cGMP-binding residues. Mutation of the third cGMP-binding site had the greatest effect on the *K*_a_(cGMP) [27]. In the same study mutation of the fourth, degenerate, site was shown to cause a decrease in the maximal enzymatic rate, but with no corresponding decrease in *K*_a_(cGMP). This led to the conclusion that this site is required for full activation, but does not bind cGMP [27]. For the *E. tenella* PKG (EtPKG) and PfPKG expressed in *T. gondii*, as well as for TgPKG, a relatively high degree of cooperativity of cGMP-dependent activation of PKG has been measured [25].

The behaviour with respect to cGMP analogues is also different. In contrast to mammalian PKGs, 8-substituted cGMP analogues are only weak activators of the EtPKG [28] and *P. falciparum* PKG [27]. Since apicomplexan PKGs were found to require binding to all three cGMP-binding domains for full activation, a restricted binding of the 8-substituted analogue to the unusual additional (third) cGMP-binding site was assumed [28]. PET-cGMP proved to be a relatively good activator of apicomplexan PKG enzymes [27,28] and this cGMP analogue may potentially be used as a molecular tool to trigger PKG activity in these systems.

There are differing reports on the stage specificity of PfPKG protein expression. Initially peak expression was documented in *P. falciparum* ring stage parasites as well as in gametocytes [8,17,25]. More recent work has found maximal asexual blood stage expression of PfPKG protein in late trophozoites and schizonts [29,30].

## 3 PfPKG mediates the initiation of *P. falciparum* gametogenesis

A small percentage of erythrocytic malaria parasites develop into gametocytes, the sexual stages, which are taken up by feeding mosquitoes and are responsible for transmission from the human to the mosquito host. As soon as the gametocytes are inside the insect, environmental changes are believed to immediately trigger gametogenesis; the differentiation to the mature gamete stage. Both male and female gametocytes change their morphology from crescent-shaped to spherical by rounding up, following which the male undergoes three rounds of genome replication to produce eight flagellated gametes, which then egress in a process termed exflagellation. This process can be stimulated *in vitro* by a temperature-decrease combined with either a rise in pH or addition of the mosquito-derived factor xanthurenic acid (XA) [31,32]. It is unclear how the stimulatory XA-signal is received by gametocytes. Pharmacological evidence suggested that increased cGMP levels could stimulate exflagellation in *P. berghei* and *P. falciparum* [33]. Furthermore a link between the stimulation of exflagellation by XA and cGMP-synthesis was identified, when addition of XA to *P. falciparum* gametocyte membrane preparations was found to stimulate GC activity [34]. This suggests that the signal for exflagellation might be transduced via a cGMP-mediated pathway [34] and that the membrane-associated GCs might directly or indirectly be involved in the reception of the XA-signal.

Since gametogenesis (and asexual blood stage development) was apparently normal in GCβ-ko parasites despite a decrease in GC activity in both *P. falciparum* [8] and *P. berghei* [9] parasites, this suggests that the critical cGMP-generating enzyme in both these stages is PfGCα [8,35,36], which was refractory to knockout approaches. Upon disruption of *PfPDEδ*, which is expressed in gametocytes, the cGMP-PDE activity was decreased by approximately 50% and the ability of gametocytes to round up and undergo exflagellation in the presence of XA was severely reduced [8]. Interestingly, it appeared that egress of the *PfPDEδ*-ko gametes from the RBC was significantly inhibited [8]. It was concluded that disruption of cGMP-PDE activity likely disturbs gametogenesis as a result of premature activation of PfPKG [8]. This indicated that a tight temporal regulation of cGMP levels by GC and PDE activities is crucial for successful sexual stage development [8].

### 3.1 A chemical genetic tool to demonstrate a role for PKG in gametogenesis

Since both PfPKG and PbPKG have proved refractory to deletion, an essential role for the enzyme in the asexual blood stages was assumed [8]. However, the lack of availability of a gene knockout has required the development of an alternative set of chemical genetic tools to study PKG function in the blood stages.

Valuable data have been generated using the PKG selective small molecules, compound 1 and compound 2 in combination with inhibitor-resistant transgenic parasites. The trisubstituted pyrrole 4-[2-(4-fluorophenyl)-5-(1-methylpiperidine-4-yl)-1*H*-pyrrol-3-yl]pyridine (compound 1) was identified in a whole-cell screen by Merck Research Laboratories and inhibits the growth of the coccidian parasites *E. tenella* and *T. gondii* both *in vitro* and *in vivo* [18,37]. In a chemical pull-down assay with compound 1, PKG was identified as a molecular target of compound 1 in *E. tenella* parasites [18]. In a subsequent medicinal chemistry effort, a more potent PKG inhibitor, the imidazopyridine compound 4-[7-[(dimethylamino) methyl]-2-(4-fluorophenyl) imidazo[1,2-*a*] pyridin-3-yl] pyrimidin-2-amine (compound 2) was developed and had greater efficacy in both *in vitro* and *in vivo* assays [38]. The selectivity of compound 1 for apicomplexan PKGs, has been attributed to interaction of a fluorophenyl group with a hydrophobic pocket that is part of the ATP-binding site. This pocket is accessible as a result of a relatively small residue threonine in the gatekeeper position, which is present in apicomplexan PKGs, but not in the mammalian isoforms [39]. An approach first used to investigate ‘off-target’ effects of the PKG inhibitors in the *T. gondii* parasite [39] was also employed to study the malarial enzyme. The gatekeeper residue of the endogenous PfPKG (threonine 618) was mutated to a bulkier glutamine. This single substitution increased the IC_50_ of compound 1 more than 3000 fold in kinase assays using recombinant enzyme. The mutant PKG was then introduced into *P. falciparum* by allelic replacement to allow investigation of the function of PKG in the various life cycle stages by comparison of mutant and wild-type parasites after treatment with compound 1 and 2 [35].

Selective chemical inhibition of PfPKG function by compound 1 and compound 2 [18] has allowed evaluation of the role of PfPKG during gametogenesis [35]. These PKG inhibitors were found to prevent both rounding up and exflagellation in *P. falciparum* gametocytes in the low micromolar range. Use of the genetically derived gatekeeper mutant of PKG confirmed that PfPKG is the primary target of compound 1 and 2 during the initiation of gametogenesis and showed that activation of gametocytes is mediated by PfPKG [35].

## 4 Function of cGMP signalling in the regulation of ookinete development and motility

Following fertilisation of the gamete in the mosquito midgut, the zygote develops into an ookinete which requires gliding motility to traverse the midgut epithelium. An essential role for cGMP signalling has been demonstrated for *P. berghei* ookinete motility. In two separate studies, the *PbGCβ* gene was disrupted [13,9]. The observations were similar: ookinetes appeared morphologically normal, but had a severe defect in gliding motility and a drastically reduced ability to invade the mosquito midgut, suggesting a role for cGMP-mediated signalling at this life cycle stage [13,9]. In accordance with this, disruption of the *PbPDEδ* gene resulted in initially normal sexual development, but instead of developing to the normal ‘banana’ shape, ookinetes gradually became spherical with a defect in motility and a greatly reduced midgut infection rate [40]. The similarities between these two phenotypes point to a requirement for tight control of cGMP levels to regulate ookinete development and motility. Low levels of cGMP as a result of a lack in GC activity, as well high cGMP levels due to a lack of PDE-mediated degradation, both could lead to a misregulation of PKG activity and cause the motility defect in ookinetes. Evidence of a direct link to PKG was obtained using compound 1, which is able to block ookinete gliding at sub- to low micromolar concentrations, indicating that the cGMP-pathway, through the actions of PKG, is crucial for ookinete motility [13]. Further evidence for this link comes from the ability of compound 1 to rescue *PbPDEδ*-ko parasites at this life cycle stage. It is hypothesised that PKG is over-activated in the *PbPDEδ*-ko parasites and therefore the compound 1-mediated inhibition of PKG activity allows normal development [13]. The *PbPDEδ*-ko phenotype is also reversed in *P. berghei* ookinetes, in which both *PbGCβ* and *PbPDEδ* were disrupted simultaneously, possibly because the decrease in cGMP-degradation can compensate for reduced cGMP-synthesis [13]. Interestingly, in the *P. berghei* parasite, a *PbCDPK3*-ko ookinete was found to mimic the reduced gliding speed phenotype of the *PbGCβ*-ko [9,36,40,41], suggesting that calcium- and cGMP-dependent pathways might overlap [13]. In accordance with this was the observation that the increased cytosolic cGMP level in the *PbPDEδ*-ko was able to rescue the phenotype of *PbCDPK3*-ko, resulting in normal motility, possibly because overstimulation of PbPKG can compensate for the loss of *PbCDPK3* signalling [13].

## 5 PbPKG function is involved in merosome release at the liver stage of *P. berghei*

After invasion of a liver cell, the malarial sporozoite replicates and eventually merozoites exit the infected hepatocyte in vesicles called merosomes, containing hundreds of parasites, which bud off the membrane. An elegant genetic knockout of *PbPKG* has been described in the liver stages of the parasite life cycle. In this approach, flippase recognition sites were introduced into the *PbPKG* locus, allowing a temperature-dependent flippase to be expressed under control of the stage-specific PbTRAP promoter. This leads to disruption of the locus only upon reaching the sporozoite stage, when infected mosquitoes were transferred to a lower temperature, in the range of 25–30 °C, prior to a blood-feed [42]. These *PbPKG*-ko sporozoites were capable of infecting hepatocytes and developing to the mature schizont stage, but merosome release was dramatically reduced [42], suggesting that similar to asexual blood stage schizonts, liver stage schizont egress is regulated through the actions of PKG.

The PKG inhibitor compound 1 was used to investigate the role of PbPKG during hepatocyte invasion by sporozoites [43]. Since *PbPKG*-ko sporozoites were capable of invading hepatocytes [42], an inhibitory effect of compound 1 on sporozoite invasion was unexpected [42], and together with the observation that *PbPKG*-ko sporozoites remained susceptible to compound 1, strongly indicated alternative targets of the inhibitor at this life cycle stage [43]. Pre-treatment of hepatocytes with the inhibitor had no effect, which pointed to the presence of compound-targets in the sporozoite, rather than the host cell. Interestingly, *E. tenella* sporozoite invasion was also prevented by compound 1 [44].

## 6 PfPKG is essential for merozoite egress from the blood stage schizont

The relevance of cGMP signalling in *Plasmodium* asexual blood stages has been assessed by studying the primary effector molecule PKG and both GC and PDE enzymes using genetic approaches. It was concluded that in asexual blood stages, GCα may be responsible for cGMP-synthesis [4,8], and PfPDEα and PfPDEβ are thought to be the primary cGMP-hydrolysing enzymes [14]. The PKG gene is refractory to deletion in both *P. falciparum* and *P. berghei* [8] and together with data from a chemical genetic approach [30], a crucial role for PfPKG in the late stages of blood stage schizont maturation became evident. The PKG inhibitor compound 1 was found to inhibit growth of *P. falciparum* asexual blood stages *in vitro* in the low μM range (IC_50_ of 2.70 μM ± 0.17) [30] and it was active against recombinant and native PfPKG enzyme in the low nanomolar range [25,35]. Parasites treated with either this inhibitor or compound 2 were able to form mature schizonts, but they were unable to rupture [30]. Direct evidence that PfPKG is the primary target of the inhibitors was generated using the T618Q gatekeeper mutant transgenic line described earlier [30]. The subcellular locations of the several schizont/merozoite proteins (merozoite surface protein 1 [PfMSP1] and rhoptry neck protein 4 [PfRON4, Pf225] [30], apical membrane antigen 1 [PfAMA1], erythrocyte binding antigen 175 [PfEBA175] and glideosome associated protein 45 [PfGAP45] [29]) involved in invasion and egress appeared normal after compound 1 treatment, suggesting a function for PfPKG very late in schizont development.

## 7 Potential molecular mechanisms regulated by PfPKG

PKG appears to have a crucial role in preparing the parasite for egress from the host cell at the asexual and sexual blood stage as well as the liver stage of the *Plasmodium* parasite. However, the downstream targets of PfPKG are unclear and the question remains as to how cGMP signalling (through PKG) is interwoven with the mechanisms known to regulate egress of apicomplexan parasites.

### 7.1 Interaction between cGMP and calcium signalling pathways

Many of the processes that are regulated by cGMP are also regulated by calcium and the two pathways are likely intersected at more than one point. A family of plant like calcium-dependent protein kinases (CDPKs) has been implicated in the processes downstream of the intracellular calcium release in *Plasmodium*. PfCDPK5 is believed to be involved in regulating egress from late blood stage schizonts, since upon inducible knockdown of PfCDPK5, rupture of mature schizonts was blocked at a very late stage, but prior to PVM and RBC membrane rupture, since these were still found intact [45]. While both PfCDPK5-knockdown, as well as pharmacological disruption of PfPKG, resulted in schizonts unable to rupture, merozoites released from PfCDPK5-knockdown schizonts were found to be viable, whereas those released from PfPKG-inhibited schizonts were not invasive [45], indicating that PfCDPK5 likely acts downstream of or in parallel to the egress cascade [45]. PfCDPK1 is also believed to have crucial functions in the maturation and segmentation of asexual *P. falciparum* blood stage parasites [46], although its position in any cascade is unclear. In *T. gondii*, calcium signalling may act together with the cGMP-dependent pathway in the regulation of microneme secretion, where PKG was thought to be acting downstream of calcium [44], since calcium ionophores were not able to reverse the PKG inhibitor-dependent block of microneme release.

Similar to the asexual schizont stage, interaction between calcium- and cGMP-dependent signalling during gametogenesis has been suggested. In the current model of gametocyte activation, XA was hypothesised to directly or indirectly activate a membrane-bound GC, resulting in increased cGMP levels which then stimulate gametogenesis through the actions of PfPKG [34]. While intracellular calcium mobilisation appeared to be required for exflagellation, raising intracellular calcium with an ionophore was found not to be sufficient to activate *P. falciparum* gametocytes [33]. It has also been observed that compound 1 blocked rounding up of gametocytes, while the calcium-chelator BAPTA-AM did not [35]. This suggested that cGMP signalling and PfPKG activity may be mediating the initiation of gametogenesis and acting upstream or independently of calcium release and likely activation of CDPK4 [35]. Interestingly, XA-dependent cGMP-synthesis in membrane fractions was inhibited by addition of calcium [34], which might indicate a direct link from calcium- to cGMP-mediated signalling. These examples illustrate that the interaction between these two signalling cascades is likely to be complex, but the molecular details are unknown.

### 7.2 Involvement of PfPKG in regulation of the egress protease cascade

While *T. gondii* tachyzoites replicate by endodyogeny, which continuously produces invasive parasites, *P. falciparum* replicates by schizogony, and as the intermediate stages are not invasive, merozoite release must be tightly controlled, since premature egress would be fatal. The mechanisms of permeabilisation of the two enclosing membranes, the PVM and host cell membrane, are still largely unresolved, although discharge of secretory organelles, the action of several proteases [47–49] and perforin-like proteins [50] have been implicated. The subtilisin-like serine protease PfSUB1 is thought to have an essential function, since a gene deletion attempt was unsuccessful, which is in line with the observation that pharmacological inhibition of PfSUB1 activity blocks egress and ablates the invasive capacity of merozoites [48]. PfSUB1 localises to dense granule-like structures, termed exonemes, which are present at the apical end of the individual merozoite [51]. Prior to egress, PfSUB1 is discharged into the PV lumen, where it is believed to mediate proteolytic activation of a family of papain-like serine rich antigen proteases (SERAs), which have further roles in completing the egress cascade [48]. Additional PfSUB1 substrates include PfMSP1, 6 and 7 [52] and proteomic and bioinformatic approaches have implicated many more [53]. Inhibition of PKG with compound 1 was found to completely block proteolytic processing of PfMSP1 [45], but the compound did not have a direct effect on PfSUB1 activity [45]. It was concluded that PfPKG is likely involved in the initiation of the proteolytic cascade that results in egress [45]. Interestingly, in contrast to schizonts treated with the PKG inhibitors, processing of PfMSP1 appeared unchanged in schizonts in which expression of PfCDPK5 had been knocked down, which also pointed to PfCDPK5 acting downstream of PfPKG or in a parallel pathway [45]. In immunofluorescence studies, PfSUB1 and PfMSP1 appeared to localise normally to the apical tip of the individual merozoites in *P. falciparum* schizonts treated with the PKG inhibitor compound 1 [29], suggesting that PfPKG is not involved in the trafficking of the protease or its substrate to their respective compartments. In *T. gondii* tachyzoites, compound 1 blocked secretion of micronemal adhesins, which the parasite uses to attach to host cells for gliding and invasion, and as a result motility and cell invasion were also inhibited at a similar efficiency to that of the protein kinase inhibitor staurosporine [44]. Since this phenotype was absent in parasites that express the gatekeeper mutated TgPKG, which is resistant to inhibition by compound 1, TgPKG was verified as the primary target of compound 1 in this process, providing clear evidence for an involvement of TgPKG in micronemal secretion [44], although the molecular mechanism is still unresolved. Investigation of a role for PfPKG in invasion is technically difficult due to the block in egress resulting from PKG inhibition. A role for the *P. falciparum* orthologue in the regulation of exoneme discharge into the PV lumen has been hypothesised [45,54]. It is of note that in *Toxoplasma*, there is little information on the potential role of TgPKG in egress, perhaps supporting the crucial role that PfPKG has in regulating the correct timing of egress in *Plasmodium*, that may not be essential in *Toxoplasma*.

## 8 Concluding remarks

Evidence suggests that PKG regulates merozoite egress from *P. falciparum* blood stage schizonts [30] and *P. berghei* liver stage schizonts [42,43]. Regulatory roles have also been found for PKG in the changes that take place in preparation for gamete egress [35], as well as in *P. berghei* ookinete development and motility [13]. The intracellular targets of PfPKG, which as a consequence are key players in the progression of the parasite life cycle, remain unknown. PKG probably has numerous downstream substrates and since it is acting at multiple stages of the parasite life cycle, it is likely to have some functionally distinct, stage-specific substrates. As advances are made in the field of phosphoproteomics and specific analyses of PKG-dependent phosphoproteomes are assembled we will be able to build a clearer picture of the consequences, at the protein level, that arise from inhibition of PKG, and how these relate to the phenotypic observations that have arisen from genetic and chemical knockdown of PKG. The reader is referred to a number of related articles on *Plasmodium* biology in this special issue [55–64].
